# Establishing an acquisition and processing protocol for resting state networks with a 1.5 T scanner: A case series in a middle-income country

**DOI:** 10.1097/MD.0000000000021125

**Published:** 2020-07-10

**Authors:** Michela Moreno-Ayure, Cristian Páez, María A. López-Arias, Johan L. Mendez-Betancurt, Edgar G. Ordóñez-Rubiano, Jorge Rudas, Cristian Pulido, Francisco Gómez, Darwin Martínez, Cesar O. Enciso-Olivera, Diana P. Rivera-Triana, Rosangela Casanova-Libreros, Natalia Aguilera, Jorge H. Marín-Muñoz

**Affiliations:** a Department of Radiology; b Department of Critical Care and Intensive Care Unit, Fundación Universitaria de Ciencias de la Salud (FUCS), Hospital Infantil Universitario de San José; c Department of Neurological Surgery, Fundación Universitaria de Ciencias de la Salud (FUCS), Hospital de San José; d Department of Biotechnology; e Department of Mathematics; f Department of Computer Science, Universidad Nacional de Colombia; g Department of Computer Science, Universidad Central; h Division of Clinical Research, Fundación Universitaria de Ciencias de la Salud (FUCS), Hospital de San José, Hospital Infantil Universitario de San José, Bogotá, Colombia.

**Keywords:** functional dynamic network connectivity, functional magnetic resonance imaging, low-to-middle income countries, neural networks, resting state networks

## Abstract

**Objective:**

The aim of this study was to characterize the capability of detection of the resting state networks (RSNs) with functional magnetic resonance imaging (fMRI) in healthy subjects using a 1.5T scanner in a middle-income country.

**Materials and methods:**

Ten subjects underwent a complete blood-oxygen-level dependent imaging (BOLD) acquisition on a 1.5T scanner. For the imaging analysis, we used the spatial independent component analysis (sICA). We designed a computer tool for 1.5 T (or above) scanners for imaging processing. We used it to separate and delineate the different components of the RSNs of the BOLD signal. The sICA was also used to differentiate the RSNs from noise artifact generated by breathing and cardiac cycles.

**Results:**

For each subject, 20 independent components (IC) were computed from the sICA (a total of 200 ICs). From these ICs, a spatial pattern consistent with RSNs was identified in 161 (80.5%). From the 161, 131 (65.5%) were fit for study. The networks that were found in all subjects were: the default mode network, the right executive control network, the medial visual network, and the cerebellar network. In 90% of the subjects, the left executive control network and the sensory/motor network were observed. The occipital visual network was present in 80% of the subjects. In 39 (19.5%) of the images, no any neural network was identified.

**Conclusions:**

Reproduction and differentiation of the most representative RSNs was achieved using a 1.5T scanner acquisitions and sICA processing of BOLD imaging in healthy subjects.

## 1 Introduction

When neurons get activated, they are provided with energy by the adjacent capillaries through a hemodynamic response, increasing regional cerebral blood flow.^[1–3]^ Consequently, a change in terms of the relative levels of oxyhemoglobin and deoxyhemoglobin is produced. Their differential magnetic susceptibilities can be detected on magnetic resonance imaging (MRI) using the blood oxygen level–dependent (BOLD) contrast imaging.^[3]^ The change in the BOLD signal is the principal landmark of functional magnetic resonance imaging (*f*MRI).^[4,5]^ As opposed to task-based *f*MRI, resting-state *f*MRI (rs*f*MRI) is acquired in the absence of a stimulus or a task. The principle of rs*f*MRI is also based on the spontaneous BOLD signal fluctuation.^[3]^

In 1995, Biswal et al found for the first time the connection and interaction that unveiled the concept of resting state networks (RSNs).^[6]^ RSNs are defined as coherent spatial fluctuations in brain activity, determined in the form of networks, identifiable when an individual is not engaged in a higher cognitive process.^[7]^ RSNs are purported to reflect the intrinsic energy demands of neuron populations that, via firing together with a common functional purpose, have subsequently wired together through synaptic plasticity.^[8,9]^ The following canonical networks have been consistently identified and reproduced in healthy subjects: default mode network (DMN), sensory/motor network (SMN), left executive control network (LECN), right executive control network (RECN), ventral attention network (VAN), auditory network (AN), cerebellar network (CBLN), and 3 visual networks (middle [MVN], lateral [LVN], and occipital [OVN]), which they have allowed physicians to establish a basis to show divergent patterns as a starting point or as biomarkers to characterize brain disorders.^[10]^ When Biswal et al described the initial reports of neural activity a standard clinical 1.5T MRI scanner was used to perform a task-based *f*MRI.^[6]^ In the last 2 decades, scanners as well as software for acquisition and processing of rs*f*MRI have been widely expanded. The exponential advent of 3T and 7T (or above) scanners have improved the analysis of normal and abnormal functional brain connectivity, with remarkable improvements in temporal and spatial resolution acquisitions.^[11]^ Even though, for clinical purposes, the availability of those hardware and software is restricted, due to high costs and lack of specialized training worldwide.

In addition, cutting edge imaging tests of the brain like the rsfMRI allow scientists to perform a thorough study of RSNs that may improve the analysis of brain connectivity in patients with disorders of consciousness (DOCs). Information in regard of neurological outcomes of patients with DOCs after traumatic brain injury (TBI) or acute stroke are scarce. Diffusion tensor imaging and rsfMRI, among other advanced imaging modalities, may improve understanding the structural and functional abnormalities in these disorders. To our knowledge, there are no studies reported in the literature with a thorough description of the RSNs with a 1.5 T scanner in low-to-middle income countries (LMICs). Our study aims to describe a simple and reproducible protocol to characterize the RSNs with *f*MRI in healthy subjects using a standard 1.5T scanner in a middle-income country.

## 2 Materials and methods

### 2.1 Clinical data and study design

A descriptive retrospective case series was conducted. Ten healthy adult volunteers were enrolled. All subjects had a nonenhanced brain MRI registered as “normal” by a former neuroradiologist (JHM). Patients were enrolled from January 2018 to March 2019. Volunteers with any of the following conditions were excluded: neurological disorders, contraindications to perform an MRI (pacemakers, metallic foreign bodies, or severe claustrophobia) or with abnormal findings on structural MRI (eg, tumors), or any condition which could not allow to perform a 20-minute study. Additionally, all subjects were also tested with the Montreal Cognitive Assessment (MoCA) test before the MRI scan. Demographic, clinical, and radiological information was collected. Authorization was requested to our Institutional Ethics Board (committee approval number 068–2016, approved in May 16^th^ of 2016) to include the information of the subjects in this study, preserving their identity both in the analysis of the information and in all images presented. All subjects provided informed consent for publication of this manuscript. This is a retrospectively analyzed study with approval by the Fundación Universitaria de Ciencias de la Salud Review Board.

### 2.2 Neuroimaging data acquisition

A 1.5T General Electric scanner was used to collect the images. One hundred and eighty multislice T2∗-weighted functional images were acquired using axial slice orientation and covering the whole brain (slice thickness = 4.5 mm without free space, matrix = 64 × 64 mm, TR = 3000 ms, TE = 60 ms, flip angle = 90 degree, and FOV = 288 × 288 mm). The three initial volumes were discarded to avoid T1 saturation effects. Finally, a structural axial T1 (slice thickness = 1 mm, GAP = 1 mm, matrix = 256 × 256 mm, TR = 670 ms, TE = 22 ms, flip angle = 20° and FOV = 250 × 250 mm) and axial T2 (slice thickness = 6 mm, GAP = 1 mm, matrix = 320 × 320 mm, TR = 6.000 ms, TE = 96 ms, flip angle = 90° and FOV = 220 × 220 mm) images were also acquired for an anatomical reference. Including complete T1 and BOLD acquisitions, a 20-minute MRI study was performed. All subjects were instructed to keep their eyes closed and not to fall asleep during the acquisition. They were not subjected to any external stimuli. For adequate processing, resting state images were obtained with T2 echo-planar imaging.^[12]^ The acquisition time ranged between 6 and 10 minutes, aiming to avoid changes in an awake state and allow sufficient images to be obtained for the identification of the RSNs.^[13]^

### 2.3 rsfMRI preprocessing

rs*f*MRI data were preprocessed using SPM8 (http://www.fil.ion.ucl.ac.uk/spm/). Preprocessing included: manual realignment, automatic realignment, coregistration of functional onto structural data, segmentation of structural data, normalization into MNI space, and spatial smoothing with a Gaussian kernel of 8 mm. The spurious variance was reduced by regression of nuisance waveforms derived of time series extracted from regions of noninterest (white matter and cerebrospinal fluid). Additionally, nuisance regressors included the BOLD time series averaged over the whole brain.^[14]^ Finally, small head motions were corrected using *ArtRepair* (http://cibsr.stanford.edu/tools/ArtRepair/).

### 2.4 Image analysis

The rs*f*MRI was decomposed into 20 independent components (ICs) with the spatial independent component analysis (sICA). Each IC of each subject was manually labeled by a former neuroradiologist (JHM), based on visual inspection of spatial maps and frequency spectra into 10 different RSNs. Those components which did not meet the assigned criteria were consequently characterized as noise, reflecting motion artifacts, physiological noise, or as partial volume effects by the cerebrospinal fluid. The 10 RSNs described in this study were: DMN, SMN, LECN, RECN, VAN, AN, CBLN, MVN, LVN, and OVN. Our previous data were published regarding functional connectivity, using these RSNs for analysis as well.^[15]^ The rs*f*MRI data were aligned, recorded, segmented, and normalized to visualize over a canonical MNI brain image.

### 2.5 Statistical analysis

Quantitative variables were calculated using median and interquartile ranges (IQRs), whereas categorical variables were demonstrated as absolute frequencies and percentages. All analytical tests were performed using the Stata statistical software (version 13).

## 3 Results

**Figure 1:**
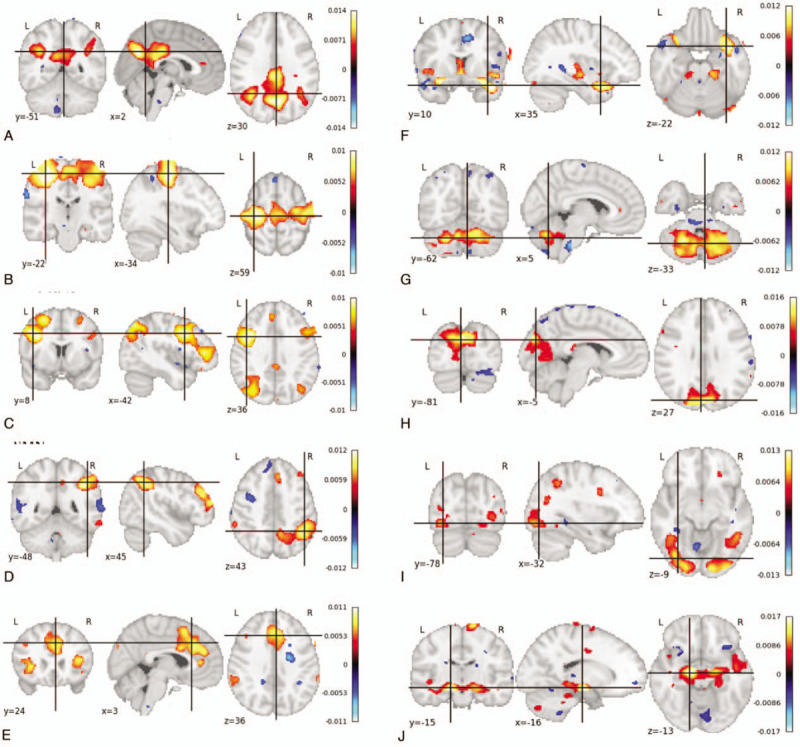
Resting state networks in healthy subjects. The canonical resting state networks are depicted in the 3 axes: (A) Default mode network, (B) motor/sensory network, (C) left executive control network, (D) right executive control network, (E) ventral attention network, (F) auditory network, (G) cerebellar network, (H) medial visual network, (I) lateral visual network, and (J) occipital visual network.

**Figure 2:**
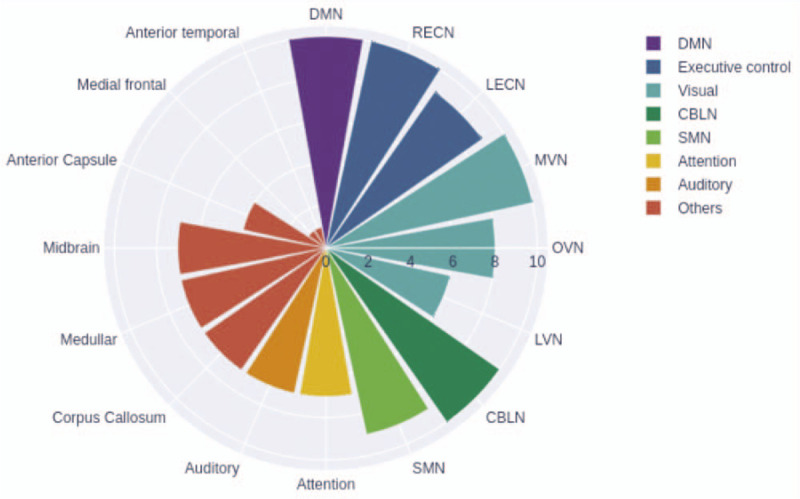
Graphic illustration of the resting state networks detected in healthy subjects. The detectability in the identification of the canonical resting state networks is demonstrated.

A demographic and clinical description of all subjects was performed and is reported in Table 1. From all patients, a total of 161 (80.5%) ICs identified the RSNs. From the 161, only 131 (65.5%) were fit for study and are detailed in Table 2. The networks that were found in all subjects were: the DMN in 26 (13%) of all documented images, the RECN in 21 (10.5%), the MVN in 11 (5.5%), and the CBLN in 16 (8%). In 90% of the subjects, the LECN and SMN were documented in 15 (7.5%) and 12 (6%) images, respectively. The OVN was documented in 8 (4%) images, and was present in 80% of the subjects. In 70% of subjects, the VAN and the AN were evidenced in 9 (4.5%) and 7 (3.5%) images, respectively. The LVN was present in 60% subjects, evidenced in 6 (3%) images. In 39 (19.5%) images, no any neural network was present at all (Figs. 1 and 2).

**Table 1:**
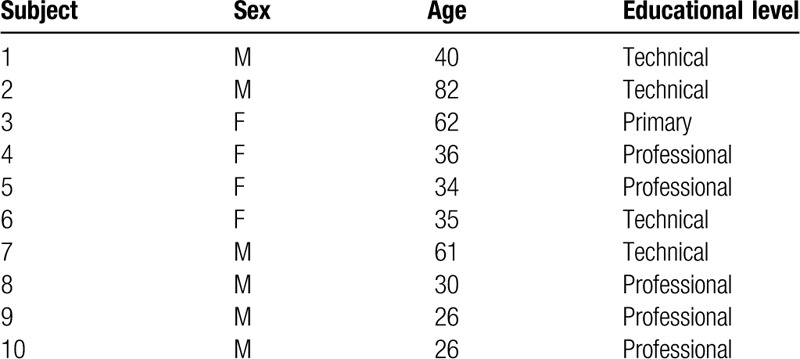
Demographic information of the subjects of the study.

**Table 2:**
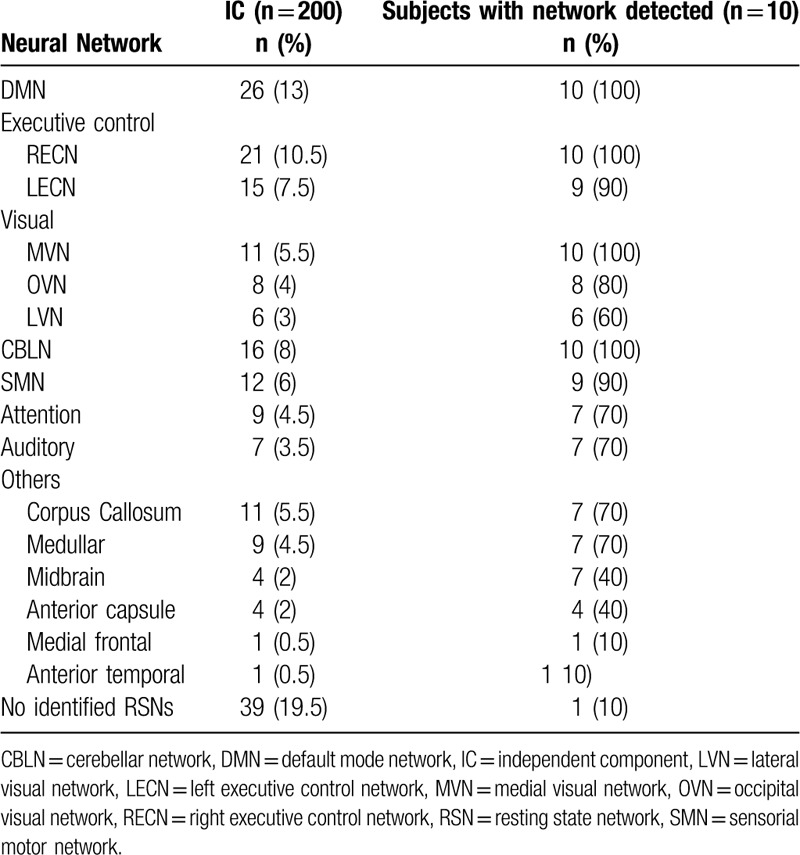
Resting state networks identified in healthy subjects.

**Figure 3:**
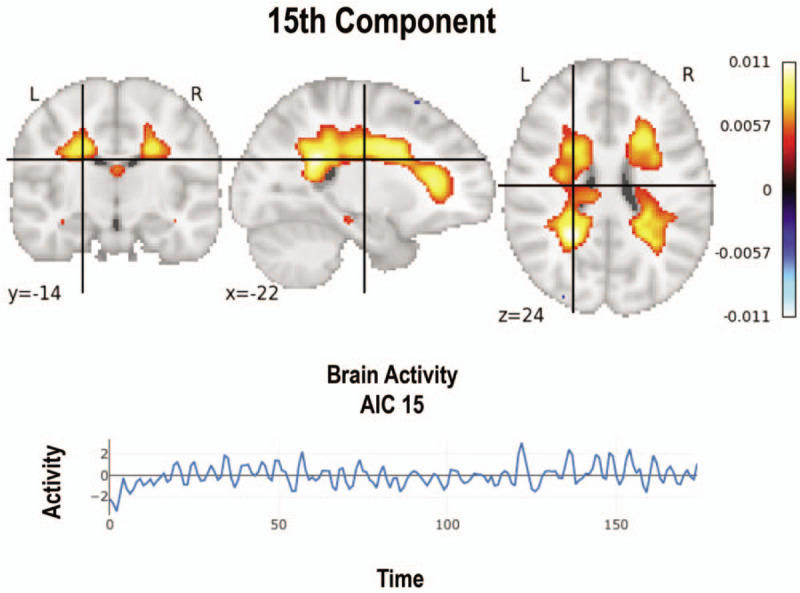
Corpus callosum signal evidenced in 7 of the studied subjects. Brain activity is demonstrated over time.

**Figure 4:**
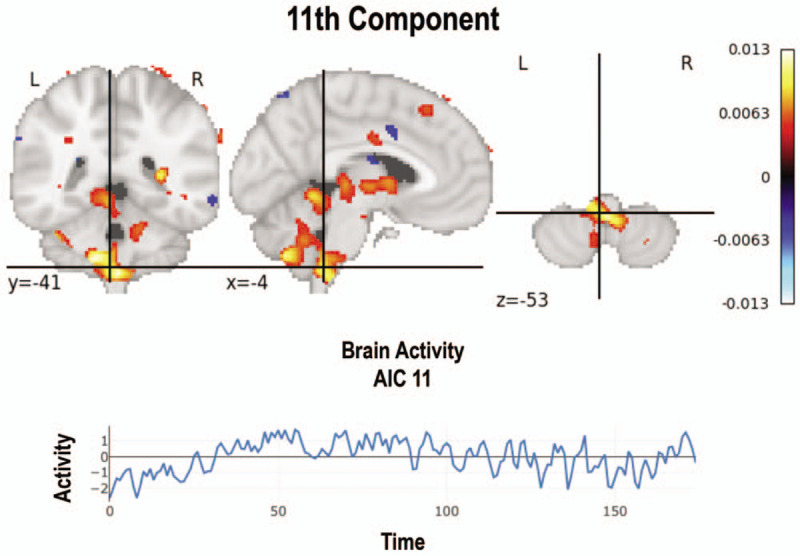
Medulla oblongata (bulb) signal evidenced in 7 of the studied subjects. Brain activity is demonstrated over time.

Other nonstudied networks were evidenced in 30 (15%) images, with a predominance of the corpus callosum (Fig. 3) and of the medulla oblongata (*a.k.a.* bulb) (Fig. 4) networks in 70% of the subjects, observed in 11 (5.5%) and 9 (4.5%) images, respectively. Likewise, other networks such as the anterior capsule and midbrain were observed in 40% of the subjects. Finally, the anterior medial and temporal frontal networks were documented in 10% of the subjects as well (Fig. 2). However, it must be noted that these latter findings could not be differentiated from any possible methodological or physiological artifact.

## 4 Discussion

### 4.1 RSNs functions

The DMN is active during memory recovery and autobiography recalling.^[16]^ This network is located in the inferior parietal cortex, precuneus, anterior and posterior cingulate, temporal mesial structures that include the dorso-lateral prefrontal cortex, thalamus, and cerebellum.^[17]^ The SMN is responsible for sensory-motor processing and includes primary somato-sensory, primary motor, premotor, and supplementary motor cortices, as well as the cerebellum. The LECN and RECN are responsible in general for the perception, selection of actions, memory, and emotional evaluation. They include bilaterally the superior, middle, and ventrolateral prefrontal cortices, and the anterior cingulate. The VAN is the network involved in the processing of attention; it is in the temporoparietal junction, in the ventral frontal cortex, the insula, and the cerebellum. The AN is responsible for auditory processing and is in the bilateral superior temporal gyrus, involving both primary and secondary auditory cortices. The CBLN network is intrinsic to the cerebellum and includes bilateral areas in cerebellar hemispheres and vermis. Finally, the visual networks (LVN, MVN, and OVN) are responsible for visual processing and are found in the lateral and medial part of the occipital lobe, involving the primary and secondary visual cortices.^[10,14]^

### 4.2 Identification of the RNSs

Our objective was to characterize the most remarkable RSNs in healthy subjects with a 1.5T MRI scanner. An automatic selection method can be used to detect neural networks and reduce subjectivity in the evaluation of the sICA. For the analysis of functional connectivity at rest, clustering algorithms have been used to divide the brain into regions (groups), which are functionally connected to each other.^[18]^ In the absence of a standard paradigm design, multivariate approaches such as the sICA are the most frequently used.^[19]^ Although the decomposition of the sICA into a *f*MRI is widely used to identify neural networks, a standard gold selection criterion for selecting networks with potential functional relevance (ie, those involved in motor function, visual processing, executive functioning, auditory processing, memory and network default mode) is still missing. We propose a rapid and useful rs*f*MRI protocol to detect the most relevant RSNs. It is important to emphasize that we also found components that could be related to networks in the corpus callosum and in the medulla oblongata in several subjects. Even though, these signals cannot be differentiated completely from any possible noise.

### 4.3 rsfMRI acquired with a 1.5T scanner and its applicability

The *f*MRI acquisitions depend on temporal physiological variables as well as thermal noise, conditioning modulations of the intensity of the image that are further related to the susceptibility weighting times and the magnetic field too.^[20]^ This has been demonstrated in previous studies that have compared 1.5T with 3T and 7T scanners, where the acquisition produced only modest increases in the temporary RSN.^[21,22]^

*f*MRI studies have been associated with the National Institutes of Health Stroke Scale score, where it has demonstrated that there is a direct relationship between the functional state of brain connectivity and the neurological outcome in patients assessed on the third day after the stroke, by the way increasing the accuracy from 84% to 94% as independent predictor of 90-day modified Rankin Scale, with the use of functional resonance studies.^[23]^ So we consider that it is important to perform more rs*f*MRI research studies with 1.5T scanners in patients with cerebrovascular and traumatic injuries aiming to further be able to predict long-term neurological outcomes. In this manuscript, we propose a rapid and useful rs*f*MRI protocol to detect the most relevant RSNs with a 1.5T MRI scanner which could be useful in future clinical neuroscience and neuropsychiatry investigations. To our knowledge, this study represents the first of his class that thoroughly describes the RSNs with a 1.5 T scanner for LMICs. We would like to enhance these findings, as they could be of interest for further research fields as well as for clinical use in LMICs. The accessibility to 3T (or above) MRI scanners remains limited worldwide, but the implementation of these technologies with 1.5T scanners may improve patient healthcare.

### 4.4 Study limitations

There are several clinical and technical limitations to this study. First is the small number of patients in whom the rs*f*MRI was performed. This algorithm for rs*f*MRI continues to evolve in the process of methodological development. rs*f*MRI is also limited by factors that include a poor sensitivity of noninvasive measurement of cerebral blood flow and the poor cortical layer specificity in blood-oxygen-level, because the regulation of blood flow is nonlocal.^[24]^ In many cases, these limitations may be related to inadequacies referable to the software level. Furthermore, this work lacks comparison to imaging data obtained with 3T (or above) scanners. The low- and middle-income economies of developing countries limit access to all specialized high-cost technologies including advanced *f*MRI. Further research is needed to clarify signal detected in the corpus callosum and in the medulla oblongata.

## 5 Conclusions

RNSs identification was achieved with a 1.5T scanner. Automatic processing of rs*f*MRI was conducted successfully as well. Technical limitations are presented, delineating the most remarkable findings in healthy subjects. Further research is needed to be applied in a clinical setting.

## Author contributions

**Conceptualization:** Michela Moreno-Ayure, Cristian Páez, María A. López-Arias, Johan L. Mendez-Betancurt, Jorge Rudas, Cristian Pulido, Francisco Gómez, Darwin Martínez, Cesar O. Enciso-Olivera, Diana P. Rivera-Triana, Rosangela Casanova-Libreros, Natalia Aguilera, Jorge H. Marín-Muñoz.

**Data curation:** Michela Moreno-Ayure, Cristian Páez, María A. López-Arias, Johan L. Mendez-Betancurt, Jorge Rudas, Cristian Pulido, Darwin Martínez, Rosangela Casanova-Libreros, Natalia Aguilera, Jorge H. Marín-Muñoz.

**Formal analysis:** Michela Moreno-Ayure, Cristian Páez, María A. López-Arias, Johan L. Mendez-Betancurt, Jorge Rudas, Cristian Pulido, Francisco Gómez, Darwin Martínez, Cesar O. Enciso-Olivera, Diana P. Rivera-Triana, Rosangela Casanova-Libreros, Natalia Aguilera, Jorge H. Marín-Muñoz, Edgar G. Ordóñez-Rubiano.

**Funding acquisition:** Cesar O. Enciso-Olivera, Jorge H. Marín-Muñoz.

**Investigation:** Michela Moreno-Ayure, Cristian Páez, María A. López-Arias, Johan L. Mendez-Betancurt, Cristian Pulido, Francisco Gómez, Darwin Martínez, Cesar O. Enciso-Olivera, Diana P. Rivera-Triana, Rosangela Casanova-Libreros, Natalia Aguilera, Jorge H. Marín-Muñoz, Edgar G. Ordóñez-Rubiano.

**Methodology:** Michela Moreno-Ayure, Cristian Páez, María A. López-Arias, Johan L. Mendez-Betancurt, Jorge Rudas, Cristian Pulido, Francisco Gómez, Darwin Martínez, Cesar O. Enciso-Olivera, Diana P. Rivera-Triana, Rosangela Casanova-Libreros, Natalia Aguilera, Jorge H. Marín-Muñoz.

**Project administration:** Cesar O. Enciso-Olivera, Jorge H. Marín-Muñoz.

**Resources:** Cesar O. Enciso-Olivera, Jorge H. Marín-Muñoz.

**Software:** María A. López-Arias, Jorge Rudas, Cristian Pulido, Francisco Gómez, Darwin Martínez.

**Supervision:** Cristian Pulido, Francisco Gómez, Darwin Martínez, Cesar O. Enciso-Olivera, Diana P. Rivera-Triana, Rosangela Casanova-Libreros, Jorge H. Marín-Muñoz.

**Validation:** Jorge Rudas, Cristian Pulido, Francisco Gómez, Darwin Martínez, Cesar O. Enciso-Olivera, Diana P. Rivera-Triana, Rosangela Casanova-Libreros, Jorge H. Marín-Muñoz.

**Visualization:** Jorge Rudas, Cristian Pulido, Francisco Gómez.

**Writing – original draft:** Michela Moreno-Ayure, Cristian Páez, María A. López-Arias, Johan L. Mendez-Betancurt, Jorge Rudas, Cristian Pulido, Natalia Aguilera, Edgar G. Ordóñez-Rubiano.

**Writing – review & editing:** Michela Moreno-Ayure, Cristian Páez, María A. López-Arias, Johan L. Mendez-Betancurt, Jorge Rudas, Cristian Pulido, Francisco Gómez, Darwin Martínez, Cesar O. Enciso-Olivera, Diana P. Rivera-Triana, Rosangela Casanova-Libreros, Natalia Aguilera, Jorge H. Marín-Muñoz, Edgar G. Ordóñez-Rubiano.
